# A novel prognostic model for lung squamous cell carcinoma based on multi-omics analysis and machine learning

**DOI:** 10.1371/journal.pone.0336792

**Published:** 2025-12-19

**Authors:** Jian Li, Zengqiang Shen, Dabei Liu, Jun Ma

**Affiliations:** Department of Thoracic Surgery, The Shanxi Provincial People’s Hospital, Shanxi, China; West China Hospital of Sichuan University, CHINA

## Abstract

Lung squamous-cell carcinoma (LUSC) is a highly aggressive malignancy with a poor prognosis. Tertiary lymphoid structures (TLS) play a crucial role in the immune response and significantly influence the efficacy of immunotherapy. However, the prognostic and immunological implications of TLS-associated molecular subtypes in LUSC remain unclear. In this study, we applied 10 multi-omics integration strategies to perform a multi-omics analysis of the mRNA expression profiles, DNA methylation, and genomic mutation data of 39 TLSs-related genes, along with long non-coding RNA (lncRNA) expression profiles, to generate integrated consensus subtypes of LUSC. Four molecular subtypes were identified: cancer subtype 1 (CS1), CS2, CS3, and CS4. We observed a significant difference in overall survival between cancer subtype 1 (CS1) and CS3. Subsequently, we identified 33 prognosis-related genes based on differential expression between CS1 and CS3, which were further refined to 20 genes using the least absolute shrinkage and selection operator (LASSO) regression algorithm, and constructed a prognostic signature termed the LUSC-Survival Prediction Index (LUSCSPI). The high-LUSCSPI group demonstrated a poor prognosis and was more likely to benefit from treatment with nine chemotherapeutic agents (shikonin, doxorubicin, CMK, S-Trityl-L-cysteine, paclitaxel, DMOG, gemcitabine, erlotinib, and crizotinib). In contrast, the low-LUSCSPI group exhibited a more favorable prognosis, with thapsigargin and cisplatin identified as promising treatment options. In conclusion, our results highlight the potential of LUSCSPI as an independent prognostic factor for LUSC. Further, the multi-omics consensus approach provides a robust foundation for prognostic stratification in LUSC patients, facilitating personalized treatment and disease management.

## 1 Introduction

Lung cancer is a main cause of cancer-related deaths worldwide. Non-small cell lung cancer (NSCLC), accounting for about 85% of lung cancer cases, is mainly sub-grouped into lung adenocarcinoma (LUAD), lung squamous-cell carcinoma (LUSC), and large-cell carcinoma [1]. Among these, LUAD compromises 50–60% of NSCLC cases, while LUSC accounts for 20–30% [2]. LUSC is characterized by high aggressiveness and a poor prognosis. Due to the lack of specific clinical manifestations in its early stages, the 5-year survival rate of LUSC is only around 10% [3,4]. The high incidence and low survival rates of LUSC impose a significant burden on society. Epidemiological studies have identified smoking, air pollution and occupational exposure as the primary risk factors for LUSC, which not only compromise patient health, but also have profound socio-economic consequences [5]. Patients with LUSC often face high medical expenses and a reduced quality of life [6]. Moreover, the diagnosis and treatment of LUSC is frequently associated with mental health challenges, such as anxiety and depression [7], highlighting the critical need for psychological support and social services.

As medical technology advances, the diagnosis and treatment of LUSC continue to evolve, encompasing surgery, radiotherapy, chemotherapy, molecular targeted therapy, and immunotherapy [8]. Since early-stage NSCLC is often localized to the lung and more resistant to chemotherapy and/or radiation, surgery remains the preferred treatment modality [8]. Radiotherapy and chemotherapy can be used independently or as an adjunct to surgery, though their efficacy is limited. Currently, platinum-based chemotherapy (typically carboplatin or cisplatin plus pemetrexed) combined with immunotherapy is the standard treatment for advanced NSCLC patients who are ineligible for approved molecular targeted therapies, regardless of histology and PD-L1 expression levels. Additionally, anti-PD-1/PD-L1 immunotherapy (pembrolizumab, atezolizumab, or cemiplimab) is the preferred approach for advanced NSCLC patients with high PD-L1 expression (≥50%) and no actionable molecular biomarkers [9]. However, a fair proportion of patients fail to derive clinical benefits from immunotherapy. Therefore, integrating multi-omics data with advanced machine learning algorithms to identify novel biomarkers for LUSC holds great promise in addressing these clinical challenges.

Tertiary lymphoid structures (TLS) have recently been identified as a distinct class of lymphoid structures, forming predominantly in chronic inflammatory diseases, autoimmune conditions, and cancers [10–12]. Composed primarily of B cells, T cells, dendritic cells, and high endothelial venules, TLS have been found to correlate with tumor prognosis and response to immunotherapy [11,13,14]. Previous studies have revealed that TLS is associated with favorable prognosis in most solid tumors, including colorectal cancer, lung cancer, breast cancer, and melanoma [15–17]. These findings highlight the role of TLS in cancer prognosis through modulation of immune infiltration. Consequently, the identification of more effective biomarkers via TLS-related molecular profiling is crucial for improving diagnostic and therapeutic strategies.

In this study, we developed an original prognosis model for LUSC based on multi-omics analysis and machine learning. We also systematically analyzed the effects of the prognosis model on tumor immunity, immunotherapy response, drug sensitivity, and tumor mutation burden in LUSC patients. In conclusion, our study provides a comprehensive assessment of the prognostic and predictive value of this model in LUSC. The results provide a theoretical foundation for refining the molecular subtypes of LUSC, facilitating personalized treatment strategies and improving disease management.

## 2 Materials and methods

### 2.1 Multi-omics data acquisition for LUSC

39 TLS-related genes (*CCL2*, *CCL3*, *CCL4*, *CCL5*, *CCL8*, *CCL18*, *CCL19*, *CCL21*, *CXCL9*, *CXCL10*, *CXCL11*, *CXCL13*, *CD200*, *FBLN7*, *ICOS*, *SGPP2*, *SH2D1A*, *TIGIT*, *PDCD1*, *CD4*, *CCR5*, *CXCR3*, *CSF2*, *IGSF6*, *IL2RA*, *CD38*, *CD40*, *CD5*, *MS4A1*, *SDC1*, *GFI1*, *IL1R1*, *IL1R2*, *IL10*, *CCL20*, *IRF4*, *TRAF6*, *STAT5A*, and *TNFRSF17*) were obtained from the study of Sautès-Fridman et al [11]. The multi-omics data for LUSC were obtained from The Cancer Genome Atlas (TCGA-LUSC) via the UCSC Xena (https://xenabrowser.net/datapages/), including mRNA expression, DNA methylation, and gene mutation data of the 39 TLS-related genes, as well as the expression of long non-coding RNAs (lncRNAs). Phenotype information of LUSC patients was retrieved from the UCSC Xena web tool, while Gene Expression Omnibus (GEO) cohorts (GSE30219) were collected using the R package “GEOquery” (version 2.66.0) [18].

### 2.2 Identification and validation of cancer subtypes (CSs) by multi-omics consensus ensemble analysis

The CSs of LUSC patients were identified using the R package “MOVICS” (version 1.0) [19]. The MOVICS package incorporates ten clustering algorithms, with their key features summarized in Table 1 [19]. The reliability and stability of the CSs were assessed using the nearest template prediction (NTP) algorithm in the R package “CMScaller” (version 2.0.1) based on the external GSE30219 cohort [20].

**Table 1:** Summary of clustering algorithms in MOVICS.

Category	Algorithm	Key Strengths
Graph-based methods	CIMLR	Integrates local and global information for optimized clustering in high-dimensional data
Graph-based methods	iClusterBayes	Leverages data features to enhance clustering performance in high-dimensional spaces
Multi-view learning	MoCluster	Clusters samples from multiple perspectives to improve stability and robustness
Collaborative clustering	COCA	Captures latent sample relationships for effective clustering analysis
Ensemble methods	ConsensusClustering	Aggregates results from multiple iterations to enhance reliability
Matrix factorization	IntNMF	Extracts latent structures efficiently, ideal for gene expression data
Low-rank approximation	LRAcluster	Processes large-scale datasets using low-rank approximation
Multi-omics integration	NEMO	Integrates diverse omics data types for comprehensive clustering
Robust clustering	PINSPlus	Mitigates noise and outliers to improve clustering accuracy
Network-based methods	SNF	Constructs and analyzes similarity networks for sample clustering

### 2.3 Development of a consensus machine learning-driven prognostic signature

Differentially expressed genes (DEGs) between CS1 and CS3 were identified using the R package “DESeq2” (version 1.38.3) [21], with a significance threshold of P < 0.05 and (|log₂FC|) > 0.28. Univariate Cox regression analysis was performed to assess the correlation between overall survival (OS) and DEGs, selecting genes with P < 0.05 for further analysis. These OS-associated genes were subsequently subjected to least absolute shrinkage and selection operator (LASSO) Cox regression using the R package “glmnet” (version 4.1–8) to construct prognostic signature for LUSC.

### 2.4 Prognosis value and verification of LUSC-Survival Prediction Index (LUSCSPI)

Kaplan–Meier survival analysis with log-rank tests was performed to assess the significant differences in OS between the high- and low-LUSCSPI groups. The same analysis was performed using the external validation set (GSE39582, GSE41258). The potential of LUSCSPI as an independent prognostic factor was evaluated by univariate and multivariate regression analyses, and further validated by constructing a nomogram.

### 2.5 Functional and pathway enrichment analyses

Biological differences between low- and high- LUSCSPI patients were assessed through functional and pathway enrichment analyses. Additionally, Kyoto Encyclopedia of Genes and Genomes (KEGG) analysis was conducted on upregulated DEGs in low- or high- LUSCSPI groups. Single-sample gene set enrichment analysis (ssGSEA) was performed using the Gene Set Variation Analysis (GSVA) package (version 1.46.0) [21] with default parameters. ssGSEA scores for Hallmark gene sets (h.all.v2022.1.Hs.symbols.gmt) and KEGG gene sets (c2.cp.kegg.v2022.1.Hs.symbols.gmt) were obtained, and differences between the low- and high-risk groups were analyzed and visualized using a heatmap (P < 0.05). The Hallmark and KEGG gene sets were downloaded from MSigDB (https://www.gsea-msigdb.org/gsea/msigdb/) [22,23].

### 2.6 Clinicopathological relevance analysis

Pearson correlation analysis was conducted to explore the relationship between LUSCSPI and various clinical characteristics, including age, P-stage, M-stage, N-stage, Ragnum Hypoxia Score, MSIsensor Score, and Buffa Hypoxia Score.

### 2.7 Difference in immune status between low- and high- LUSCSPI groups

The ssGSEA scores of 28 immune cell types were assessed using the R “GSVA” package (version 1.46.0). Differential expression of immune biomarkers, immune checkpoints, and human leukocyte antigen (HLA)-related genes between low- and high-risk patients was further evaluated.

### 2.8 Prediction of patient responses to chemotherapy

The sensitivity of LUSC patients to 11 chemotherapeutic agents, including thapsigargin, shikonin, doxorubicin, CMK, s-trityl-l-cysteine, paclitaxel, DMOG, cisplatin, gemcitabine, erlotinib, and crizotinib, was estimated using the R package “pRRophetic” (version 0.5) based on data from the Genomics of Drug Sensitivity in Cancer (GDSC) database (https://www.cancerrxgene.org/) [24]. Wilcoxon signed-rank tests were used to compare the IC50 values between low- and high-LUSCSPI patients. A high IC50 value indicates lower insensitivity to the drug.

### 2.9 Mutation status analysis of LUSCSPI

The oncoprint of 20 genes in LUSCSPI, along with frequently mutated cancer-related genes, was compared between the high- and low-LUSCSPI groups using data obtained from the cBioPortal database (https://www.cbioportal.org/) [25]. Pearson’s correlation analysis was conducted to assess the correlation coefficients between four pan-cancer-related genes and LUSCSPI.

### 2.10 Statistical analysis

Statistical analyses were conducted using R software (version 4.1.1). We performed Kaplan-Meier survival analyses with log-rank tests to assess significant differences in OS between the two groups. Data normality was evaluated using the Kolmogorov-Smirnov test. For data that followed a normal distribution, we utilized the Student’s t-test to compare gene expression differences between the two groups. In contrast, the Wilcoxon test was employed to evaluate drug sensitivity across high- and low-LUSCSPI groups. Additionally, Pearson’s correlation analysis was conducted to assess the relationship between LUSCSPI and clinicopathological features, drug sensitivity, four pan-cancer-related genes, and other cancer-related gene expression, allowing for the calculation of correlation coefficients. Statistical significance was defined as P < 0.05.

## 3 Results

### 3.1 Characterization of multi-omics-based subtypes of LUSC

**Fig 1:**
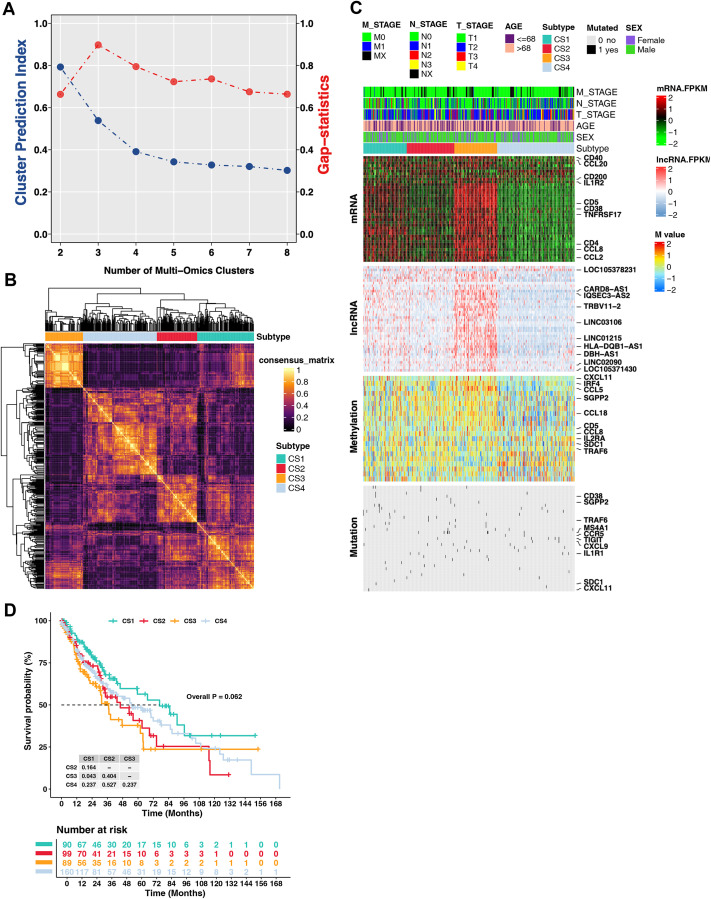
Multi-omics-based classification of cancer subtypes (CSs) in lung squamous cell carcinoma (LUSC) patients. (A) Determination of clustering coefficients based on the Cluster proportion index (blue line) and Gaps-statistics (red line) in the TCGA-LUSC cohort. (B) Consensus clustering heatmap generated using 10 multi-omics ensemble clustering algorithms. (C) Comprehensive heatmap depicting the four CSs derived from multi-omics consensus ensemble analysis, integrating mRNA expression, lncRNA expression, DNA methylation, and gene mutation data. (D) Kaplan-Meier overall survival (OS) analysis comparing the four subtypes in the TCGA-LUSC cohort.

LUSC is characterized by significant molecular heterogeneity, posing challenges for precise prognosis prediction. To mitigate this heterogeneity, we employed a multi-omics approach to delineate consensus prognosis-related molecular subtypes of LUSC. Cluster analysis was performed based on mRNA expression, gene mutation, and gene methylation data of the 39 TSL-related genes, as well as lncRNA expression data, ultimately resulting in the identification of four subtypes: CS1, CS2, CS3, and CS4 (Fig 1 A-1B). A comprehensive heatmap of consensus ensemble subtypes revealed notable differences among the four subtypes, with subtype CS3 showing relatively upregulated mRNA and lncRNA expression. The top 10 mRNA features associated with subtyping included *CD40*, *CCL20*, *CD200*, *IL1R2*, *CD5*, *CD38*, *TNFRSF17*, *CD4*, *CCL8*, and *CCL2*, while the top 10 mutations with a high impact on subtyping included *CD38*, *SGPP2*, *TRAF6*, *MS4A1*, *CCR5*, *TIGIT*, *CXCL9*, *IL1R1*, *SDC1*, and *CXCL11*. LOC105378231, CARD8-AS1, IQSEC3-AS2, TRBV11−2, LINC03106, LINC01215, HLA-DQB1-AS1, DBH-AS1, LINC02090, and LOC105371430 were the representative lncRNAs, while *CXCL11*, *IRF4*, *CCL5*, *SGPP2*, *CCL18*, *CD5*, *CCL8*, *IL2RA*, *SDC1*, and *TRAF6* were the genes associated with the methylated sites (Fig 1C). When comparing the clinical prognosis outcomes among the four subtypes, patients in CS1 exhibited significantly better OS than those in CS3 (P < 0.05) (Fig 1D).

### 3.2 Distinct molecular landscape of the four LUSC CSs in immune environment, metabolic pathways, and chromatin remodeling profiling

The four CSs were systematically compared in terms of tumor microenvironment, metabolism, and chromatin remodeling. Differences in immune checkpoint gene expression and immune cell infiltration were analyzed. Heatmap analysis showed that the expression of *IL27RA*, *L27*, *CTLA4*, *PDCD1*, *CD274*, and *BCL2* were significantly upregulated in CS3 patients. This upregulation was accompanied by the activation of multiple immune cells, such as activated B cell, activated CD8 T cell, activated CD4 T cell, activated dendritic cell, and natural killer cell (S1A Fig). Moreover, heatmap analysis of ssGSEA scores for KEGG pathways revealed distinct metabolic profiles among the four subtypes. Specifically, CS3 exhibited enrichment in pathways such as linoleic acid metabolism, alpha-linolenic acid metabolism, ether lipid metabolism, fatty acid biosynthesis, arachidonic acid metabolism, and histidine metabolism. In contrast, CS4 was related to ascorbate and aldarate metabolism, pentose and glucuronate interconversions, porphyrin and chlorophyll metabolism, steroid hormone biosynthesis, retinol metabolism, drug metabolism, metabolism of xenobiotics by cytochrome P450, and pyrimidine metabolism. Additionally, both CS1 and CS2 were enriched in amino sugar and nucleotide sugar metabolism, as well as fructose and mannose metabolism (S1B Fig). To further investigate transcriptomic differences, we analyzed potential regulators associated with chromatin remodeling and transcription factors (TFs). Notably, several chromatin remodeling regulators, including *KAT2A*, *HDAC10*, *SIRT7*, *HDAC8*, *SIRT5*, *CARM1*, *SMARCA4*, and *SMARCD1* were upregulated in CS4. Regarding TFs, ESR1, GATA3, STAT3, PGR, GATA6 were enriched in CS3 but downregulated in CS1 and CS4 (S1C Fig). These observed differences in TFs and regulatory activity patterns suggest distinct underlying chromatin remodeling mechanisms among the subtypes. Based on differential expression analysis between subtypes, genes specifically upregulated in each subtype were selected as classifiers and validated using an external GEO cohort. The results confirmed the stability of the identified subtypes, aligning with the clustering patterns observed in Fig 1B (S1D-S1E Figs).

### 3.3 Construction and verification of LUSC-related risk signature

**Fig 2:**
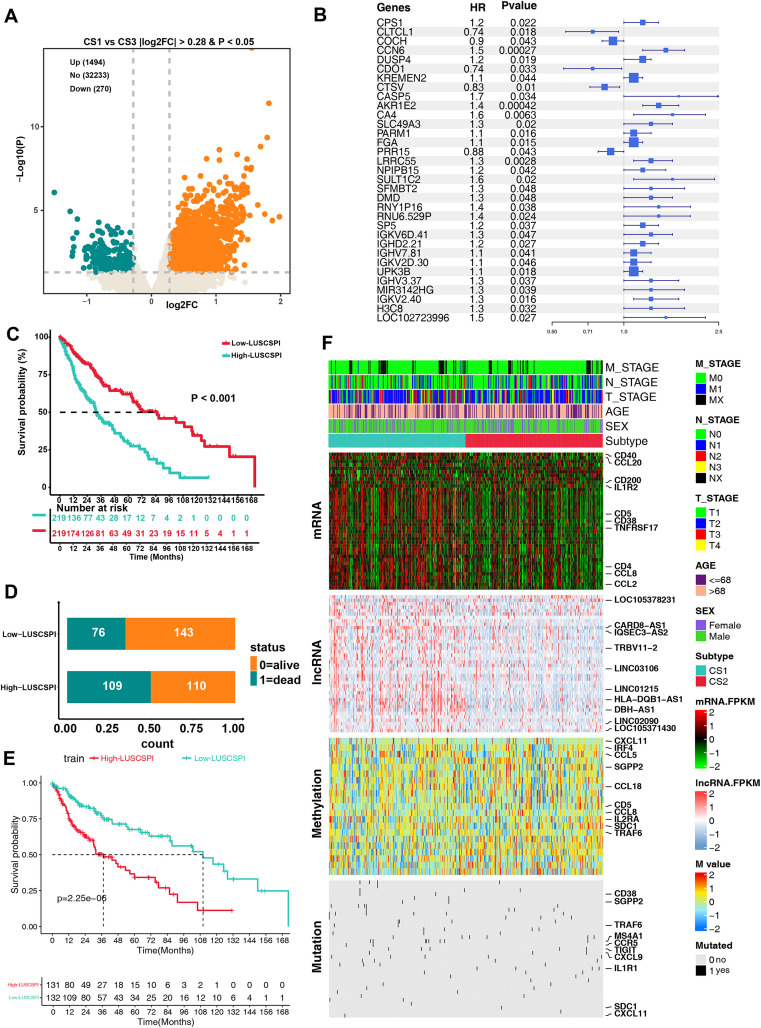
Development of the LUSC prognostic signature consisting of 20 genes. (A) Volcano plot demonstrating the upregulated and downregulated DEGs between CS1 and CS3 (P < 0.05 and |log2foldchange| > 0.28) (B) Univariate Cox regression analysis of the DEGs between CS1 and CS3 revealed 33 prognosis-related genes (P < 0.05). (C) Kaplan-Meier survival analysis of OS in the TCGA-LUSC cohort, comparing groups defined by the LUSCSPI classifier. (D) Patient survival status counts in the low- and high-LUSCSPI groups. (E) Kaplan-Meier survival validation in the TCGA-LUSC training cohort. (F) Comprehensive heatmap analysis of multi-omics data (mRNA, lncRNA, DNA methylation, and gene mutations) between low- and high-LUSCSPI groups.

Due to the significant OS difference between CS1 and CS3, a differential analysis was performed between these two groups. A total of **1494** upregulated and **270** downregulated DEGs were identified in CS1 (Fig 2A). To further evaluate the prognostic significance of these DEGs, univariate Cox regression analysis was conducted, revealing 33 prognosis-related genes (P < 0.05) (Fig 2B). Subsequently, we applied the least absolute shrinkage and selection operator (LASSO) regression algorithm to refine the selection and identify key candidates for constructing the risk score model. As a result, a prognostic signature for LUSC was established, comprising 20 genes. The details of these genes, along with their corresponding coefficients, are provided in S1 Table. The risk score, termed LUSC-Survival Prediction Index (LUSCSPI), was developed to stratify LUSC patients into high-LUSCSPI or low-LUSCSPI groups with distinct OS outcomes. As visualized in the survival curves, significant differences were observed in clinical outcomes between low- and high-LUSCSPI groups, with high-LUSCSPI being significantly negatively associated with OS (P < 0.001) (**Fig 2C**-2E). We further examined the distribution of the multi-omics data between the two groups. *CD20*, *CCL20*, *CD200*, *IL1R2*, *CD5*, *CD38*, *TNFRSF17*, *CD4*, *CCL8*, *CCL2*, LOC1065378231, CARD8-AS1, IQSEC3-AS2, LINC03106, LINC01215, HLA-DQB1-AS1, DBH-AS1, LINC02090, and LOC105371430 were identified as the top 20 OS-related factors in the transcriptome (mRNAs and lncRNAs) (Fig 2F). *CXCL11*, *IRF4*, *CCL5*, *SGPP2*, *CCL18*, *CD5*, *CCL8*, *IL2RA*, *SDC1*, and *TRAF6* were the top 10 OS-related factors in DNA methylation (Fig 2F). Moreover, *CD38*, *SGPP2*, *TRAF6*, *MS4A1*, *CCR5*, *TIGIT*, *CXCL9*, *IL1R1*, *SDC1*, and *CXCL11* were the top 10 OS-related factors of gene mutations (Fig 2F). These results highlight distinct differences between low- and high- LUSCSPI groups.

### 3.4 Correlation analysis with clinicopathological features revealed LUSCSPI as an independent prognostic factor for LUSC

**Fig 3:**
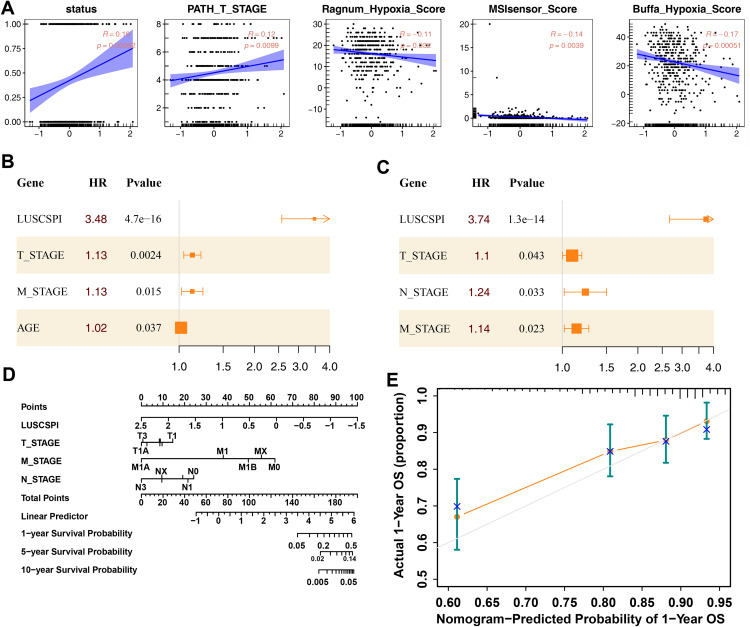
Correlation analysis with clinicopathological features revealed LUSCSPI as an independent prognostic factor for LUSC. (A) Correlation analysis between LUSCSPI and clinicopathological features, including status, T-stage, Buffa Hypoxia Score, Ragnum Hypoxia Score, and MSIsensor Score. (B) Univariate Cox regression analysis of LUSCSPI and clinicopathological features (T-stage, M-stage, age). (C) Multivariate Cox regression analysis of LUSCSPI and clinicopathological features (T-stage, N- stage, M-stage). (D) A comprehensive nomogram based on LUSCSPI, T-Stage, N- Stage, and M-Stage to predict the 1-, 5-, and 10-year OS in LUSC patients. (E) Calibration curve of LUSCSPI for predicting 1-year OS.

Next, we performed a correlation analysis to investigate the relationship between LUSCSPI and the clinicopathological features of LUSC. The results showed that LUSCSPI was significantly positively correlated with status and T-stage, while it exhibited negative correlations with Buffa Hypoxia Score, Ragnum Hypoxia Score, and MSIsensor Score (Fig 3A). To assess the independent prognostic value of LUSCSPI in patients, we conducted both univariate (Fig 3B) and multivariate (**Fig 3C**) Cox regression analyses. The results indicated that LUSCSPI could serve as an independent prognostic factor for LUSC, regardless of clinicopathological factors such as age, T-stage, M-stage, N-stage (hazard ratio [HR]: 3.7374, P < 0.001). Based on these findings, we constructed a prognostic nomogram incorporating LUSCSPI, T-stage, M-stage, and N-stage in the TCGA-LUSC cohort to predict the 1-, 5-, and 10-year OS probabilities **(Fig 3D)**. The calibration plot further confirmed that the nomogram accurately predicts 1-year OS **(Fig 3E)**. These results highlight the robust predictive performance of LUSCSPI, demonstrating its potential as an independent prognostic factor or in combination with existing clinicopathological features. Comprehensive data on clinicopathological characteristics are provided in S2 Table.

### 3.5 High- and low-LUSCSPI patients showed distinct immune cell composition and immune modulator expression

**Fig 4:**
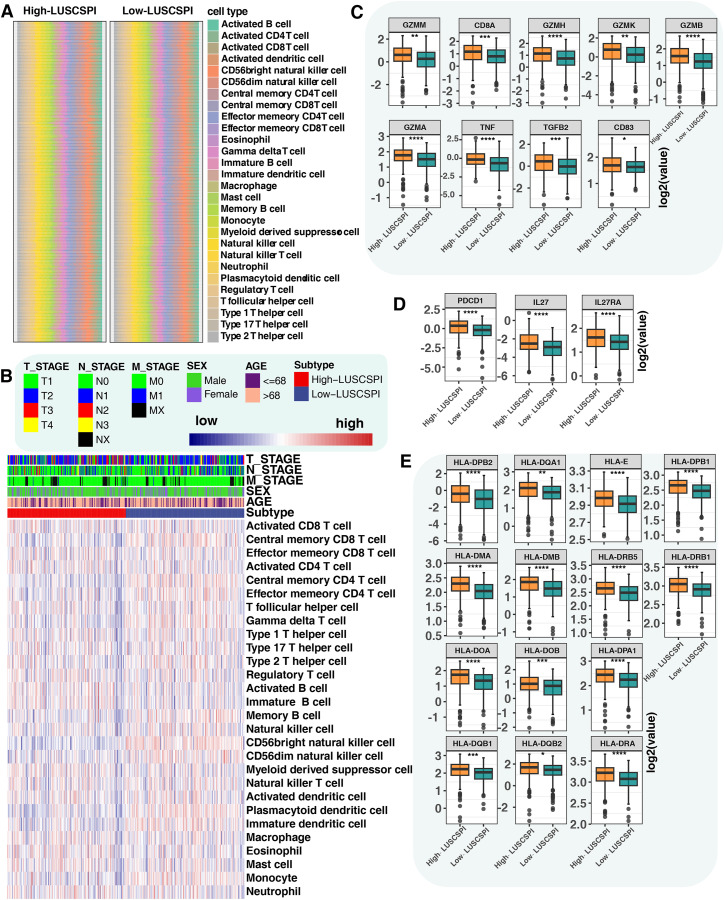
High- and low-LUSCSPI patients showed distinct immune cell composition and immune modulator expression. (A) Stacked bar chart showing the proportions of 28 immune cell types in high- and low-LUSCSPI patients. (B) Heatmap demonstrating the abundance of 28 immune cell types based on the ssGSEA algorithm between high- and low-LUSCSPI patients. (C-E) Boxplots comparing the expression levels of immune-related (C), immune checkpoints-related (D), and HLA-related **(E)** genes between high- and low-LUSCSPI patients. *P < 0.05; **P < 0.01; ***P < 0.001. ****P < 0.0001.

To investigate the immune landscape in different risk groups, we assessed the proportion of 28 immune cell types using the ssGSEA algorithm. The distribution of immune cells in low- and high-risk groups is presented in stacked bar plots (Fig 4A). Notably, several immune cells exhibited distinct abundance patterns between the two groups. Specifically, the low-LUSCSPI group showed significantly higher abundances of CD56dim natural killer cells, effector memory CD4 T cells, memory B cells, CD56bright natural killer cells, immature dendritic cells, plasmacytoid dendritic cells, central memory CD8 T celsl, gamma delta T cells, monocytes, central memory CD4 T cells, and natural killer cells. Conversely, high-LUSCSPI patients showed a larger proportion of eosinophils, activated B cells, immature B cells, activated CD8 T cells, and activated CD4 T cells (Fig 4B, S3 Table). These findings may suggest that LUSC patients with low LUSCSPI have higher levels of immune cell infiltration, and that LUSCSPI may be associated with prognosis through increased levels of activated CD8 T cells and activated CD4 T cells, which are indicative of a worse OS outcome. Considering the importance of immune modulators in anticancer immunity, we investigated the expression levels of immune-related, immune checkpoints-related, and human leukocyte antigen (HLA)-related genes between the two risk groups. The high-LUSCSPI group showed higher expression of genes such as *GZMM*, *GZMA*, *TNF*, *PDCD1*, *IL27*, and *HLA-DPB2* (Fig 4C-4E, S4 Table). The results suggest that LUSCSPI may be associated with the expression of immune modulators, supporting its potential as an immune therapeutic biomarker.

### 3.6 KEGG and ssGSEA enrichment analyses revealed biological pathway differences between low- and high-LUSCSPI groups

**Fig 5:**
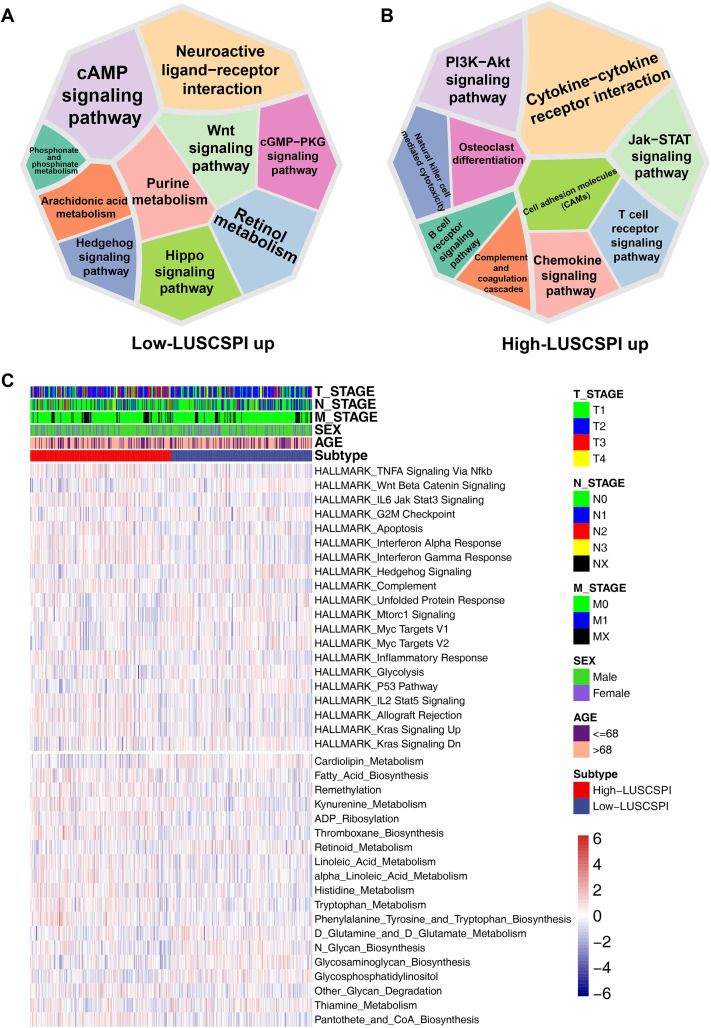
KEGG and ssGSEA enrichment analyses revealed biological pathway differences between low- and high-LUSCSPI groups. (A) Kyoto Encyclopedia of Genes and Genomes (KEGG) enrichment analysis of upregulated DEGs in low-LUSCSPI patients (P ≤ 0.05, FC > 2). (B) KEGG enrichment analysis of upregulated DEGs in high-LUSCSPI patients (P ≤ 0.05, FC > 2). (C) Heatmap displaying metabolism-related pathways between high- and low-LUSCSPI patients using the ssGSEA algorithm. The top panel shows the enriched levels of metabolic pathways based on the HALLMARK database, and the bottom panel shows the enrichment level of metabolic pathways based on the KEGG database.

To investigate the biological differences in both low- and high-LUSCSPI groups, we conducted KEGG enrichment analysis. The results indicated that the DEGs upregulated in low-LUSCSPI group were mainly enriched in neuroactive ligand-receptor interaction, wnt signaling pathway, and cAMP signaling pathway (Fig 5A). In contrast, the high-LUSCSPI group showed enrichment in immune-related pathways, including natural killer cell mediated cytotoxicity, Jak-STAT signaling pathway, cell adhesion molecule, B cell receptor signaling pathway, T cell receptor signaling pathway, complement and coagulation cascades, and chemokine signaling pathway (Fig 5B). We then conducted ssGSEA based on the Hallmark and KEGG gene sets. As shown in the heatmap, most of the metabolism-related pathways, such as linoleic acid metabolism, alpha linoleic acid metabolism, phenylalanine, tyrosine and tryptophan biosynthesis, fatty acid biosynthesis, pantothete and CoA biosynthesis, histidine metabolism, thromboxane biosynthesis, kynurenine metabolism, and tryptophan metabolism, were activated in the high-LUSCSPI group. Additionally, immune-associated pathways, including HALLMARK interferon alpha response, HALLMARK interferon gamma response, HALLMARK_IL2 Stat5 signaling, HALLMARK complement, HALLMARK IL6 Jak Stat3 signaling, and HALLMARK inflammatory response, were enriched in the high-LUSCSPI group (Fig 5C). These results suggest that LUSCSPI, as a novel biomarker for LUSC, may play a role in crucial immune-related signaling pathways.

### 3.7 Low-LUSCSPI group showed higher mutation frequencies

Next, we analyzed the correlation between cancer-related genes and LUSCSPI. Several genes, such as *FGFR2*, *IDH1*, *PIK3CA*, *KRAS*, *EGFR*, *MET*, *NF1*, *NTRK2*, and *SMARCB1*, were found to be significantly negatively correlated with LUSCSPI. In contrast, *ROS1*, *IDH2*, *KIT*, *NTRK1*, and *TSC2*, exhibited a significant positive correlation with LUSCSPI (S3 Fig). Increased expression of cancer-related genes and higher somatic mutation rates are associated with enhanced antitumor immunity. To explore potential oncogenic drivers in LUSC, we examined the distribution of the 20 genes in the LUSCSPI risk score model, focusing on those with somatic hypermutation. S2 Fig in S2A depicted the mutation profiles of these genes in the LUSCSPI risk score model, comparing the high- and low-LUSCSPI groups. Interestingly, the low-LUSCSPI group exhibited a higher somatic mutation frequency than the high-LUSCSPI group, with genes such as *CLTCL1*, *CPS1*, *PARM1*, *FGA*, *H3C8*, *AKR1E2*, *CA4*, *SLC49A3*, *SULT1C2*, *CTSV*, and *CDO1* being more frequently mutated in the low-LUSCSPI group. Moreover, the mutation frequency of most frequently mutated LUSC genes, including *PIK3CA*, *EGFR*, *ROS1*, and *NRAS*, were also higher in the low-LUSCSPI patients than the high-LUSCSPI patients (S2 Fig in S2B ). In conclusion, the majority of the highly mutated genes were found in the low-LUSCSPI group.

### 3.8 Screening of potential therapeutic drugs for LUSC

**Fig 6:**
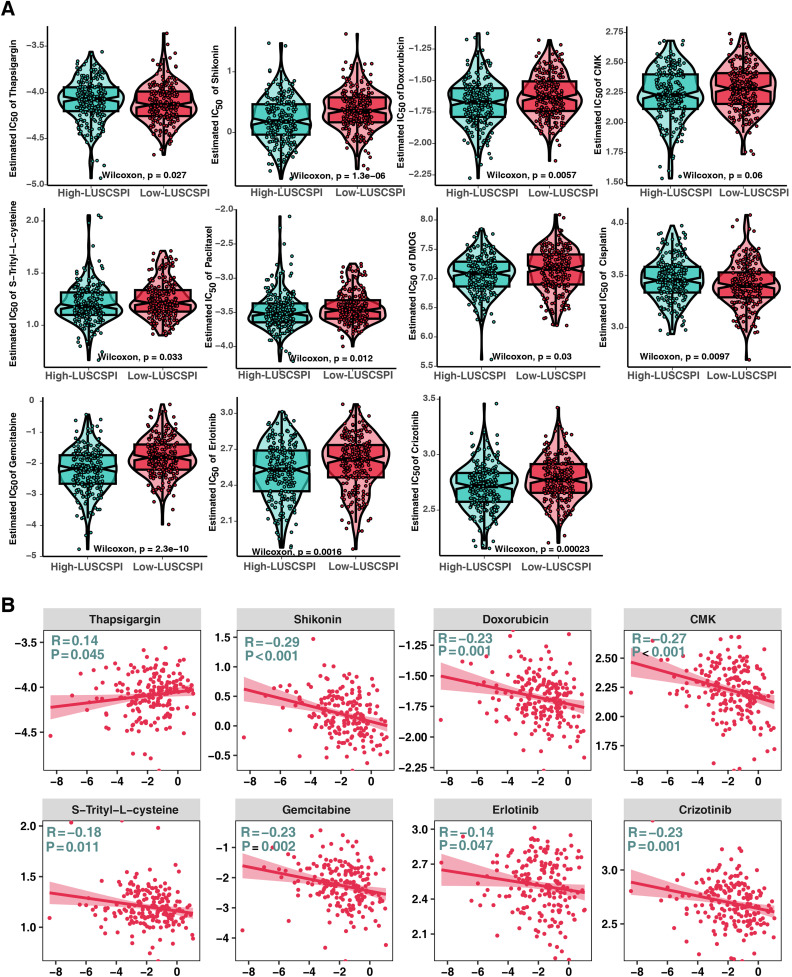
Potential therapeutic agents for high- and low-LUSCSPI patients. (A) Differences in drug sensitivity to 11 chemotherapeutic agents between high- and low-LUSCSPI patients. (B) Correlation analysis between drug sensitivity and LUSCSPI.

Given that chemotherapy is the current standard clinical treatment for patients with LUSC, we used the “pRRophetic” package to estimate patient sensitivity to 11 chemotherapeutic agents, including thapsigargin, shikonin, doxorubicin, CMK, S-Trityl-L-cysteine, paclitaxel, DMOG, cisplatin, gemcitabine, erlotinib, and crizotinib. The result indicted that the IC50 values of nine drugs (shikonin, doxorubicin, CMK, S-Trityl-L-cysteine, paclitaxel, DMOG, gemcitabine, erlotinib, and crizotinib) were lower in patients with high-LUSCSPI scores. However, the IC50 values of only thapsigargin and cisplatin were lower in low-LUSCSPI patients (Fig 6A). The relatively low estimated IC50 values of these chemotherapeutic agents in the high-LUSCSPI group may indicate that these patients might be more sensitive to these drugs. Moreover, Pearson correlation analysis between LUSCSPI and IC50 values revealed predominantly negative correlations (P < 0.05) (**Fig 6B**). These findings demonstrated that thapsigargin and cisplatin could be promising therapeutic options for low-LUSCSPI patients.

## 4 Discussion

Recently, targeted therapies and immunotherapy have provided new hope for tumor treatment. The selection of treatment largely depends on tumor stage. However, the highly heterogeneous nature of LUSC complicates traditional staging methods, contributing to the limited treatment options and poor prognosis for LUSC patients. Consequently, there is an urgent need to identify molecular subtypes of LUSC and provide more precise, individualized treatment for the patients. Numerous biomarkers, such as specific genes, mRNA/lncRNA/miRNA signatures, and DNA methylation markers, have been identified and utilized in clinical settings [26,27]. TLS, a class of lymphoid structures, have recently been identified to form in a variety of chronic inflammatory diseases, autoimmune diseases, and cancers [11,12]. Studies have shown that TLS significant impacts the prognosis of many solid tumors, such as intrahepatic cholangiocarcinoma [27], endometrial cancer [28], oral cavity cancer [29], lung cancer [30], and renal clear cell carcinoma [31]. It may play a direct role in the tumor immune response [32]. Therefore, the identification of more effective markers through TLS-related molecular typing is crucial for early diagnosis and prognosis evaluation.

In this study, relevant data on TLS-related genes were obtained for multi-omics analysis of LUSC. Among these genes, CD40 is an important immunomodulatory molecule, which promotes anti-tumor immune responses by regulating the function of immune cells [33,34]. CCL20, also known as intestinal epithelial chemoattractin (MIP-3α), is a chemokine that promotes tumor development and represents a potential therapeutic target [35–37]. CD38 is a transmembrane glycoprotein that functions as an immunosuppressive factor within the microenvironment of solid tumors. Targeting CD38 in solid tumor therapy is expected to achieve its inhibitory effects on immune cells [38]. Inhibition of CD38 has been reported to alleviate tumor-induced immunosuppression by reducing adenosine production [39]. Interleukin-1 receptor type II (IL-1R2), acting as a negative regulator of the IL-1 system, plays a key role in various inflammatory diseases and cancers [40]. In our study, we found that most TLS-related genes, such as *CD40*, *CCL20*, *CD200*, *IL1R2*, *CD38*, exhibited alterations in both expression and mutation in LUSC patients. Similar observations have been reported in previous studies. For instance, *CD40* expression has been shown to be downregulated in both LUSC and LUAD [41]. Another study revealed that the mRNA expression of *CCL20* was upregulated in LUSC and LUAD tissues [42]. *CD200* positivity was observed in 29.7% of patients with NSCLC and 33.3% of patients with lung large cell neuroendocrine carcinoma (LCNEC) [43]. Furthermore, immunohistochemical analysis revealed higher *IL1R2* expression in tumor tissues of LUAD patients, with lower *IL1R2* levels being associated with better prognosis [44]. Moreover, *CD38* expression has been reported to correlate with survival outcomes and immune infiltration levels in NSCLC [45]. These gene alterations may contribute to tumorigenesis and the modulation of immune responses.

In this research, ten clustering algorithms were integrated to obtain four prognostic subtypes with different characteristics based on TLS-related multi-omics data. Differences in immune infiltration profiles and metabolic pathways were observed among the four subtypes. For example, CS3 was characterized by the activation of various immune cells, such as activated B cells, activated CD8 T cells, activated CD4 T cells, activated dendritic cells, and natural killer cells. In contrast, CS4 was related to ascorbate and aldarate metabolism, pentose and glucuronate interconversions, porphyrin and chlorophyll metabolism, steroid hormone biosynthesis, retinol metabolism, drug metabolism, metabolism of xenobiotics by cytochrome P450, and pyrimidine metabolism. These pathways are crucial for maintaining cellular redox homeostasis and detoxification. In the context of CS4, the upregulation of these pathways may reflect the enhanced ability to counteract oxidative stress and detoxify harmful metabolites in the subtype, which is essential for tumor cell survival and proliferation [46,47]. Furthermore, we constructed a prognostic signature LUSCSPI, which was demonstrated to have a strong association with immune microenvironment of LUSC. Immune cells, such as activated B cell, immature B cell, activated CD8 T cell, and activated CD4 T cell were higher in the high-LUSCSPI group with an upregulated expression of immune checkpoint genes. Through the ssGSEA, we found that various vital immune-related signaling pathways were significantly activated in the high-LUSCSPI group. Inhibitory immune checkpoints have been reported to be fundamental in cancer progression and the suppression of the immune system [48]. Immune checkpoint inhibitors have emerged as a promising approach for cancer therapy [49]. For instance, immune checkpoint inhibitor therapy targeting PD-L1/PD-1 has been shown to be particularly effective in NSCLC treatment [50]. In our study, we observed higher *PDCD1* expression in the high-LUSCSPI group compared with the low-LUSCSPI group. Based on these results, we propose that the high-LUSCSPI group, characterized by a high proportion of B cells and CD8 + T cells, along with elevated *PDCD1* expression, may be more responsive to immunotherapy.

Since chemotherapy remains a standard treatment for lung cancer, we explored the chemosensitivity between the high- and low-LUSCSPI groups based on IC50 values. High-LUSCSPI patients exhibited increased sensitivity to nine common drugs (shikonin, doxorubicin, CMK, S-Trityl-L-cysteine, paclitaxel, DMOG, gemcitabine, erlotinib, and crizotinib), potentially leading to more favorable therapeutic outcomes. The primary mechanisms of these drugs involve the inhibition of cell proliferation or the promotion of apoptosis [51–55]. For instance, shikonin has been reported to induce apoptosis in cancer cells through Ras/MAPK and PI3K/AKT Pathways [56]. Doxorubicin, a widely-used anthracycline, intercalates into DNA, inhibits topoisomerase II, and ultimately causes DNA damage-induced apoptosis [57]. In contrast, thapsigargin and cisplatin may be effective treatment options for low-LUSCSPI patients. Previous research has revealed that thapsigargin treatment can effectively inhibit the proliferation of prostate cancer cells [58]. Moreover, cisplatin, a widely used cancer chemotherapy drug [59], primarily targets malignant tumors, such as ovarian cancer [60], lung cancer [61,62], breast cancer [63], and head and neck cancer [64]. Its therapeutic effects are mainly attributed to its ability to interfere with DNA replication and repair processes in tumor cells, thereby preventing cancer cell proliferation and growth [65]. Therefore, LUSC subtyping may help identify patient subgroups that could benefit from either immunotherapy or chemotherapy.

In conclusion, our study constructed a risk model compromising 20 genes, which was proved to be an independent and reliable predictor of prognosis for LUSC. This model not only aids in the clinical risk stratification of LUSC but also provides new perspectives for personalized and precise treatment strategies for LUSC patients. However, this study utilized publicly available datasets, resulting in the absence of sample validation; therefore, further experimental validation is needed to confirm and elucidate the exact regulatory mechanism of LUSCSPI in LUSC.

## Supporting information

S1 Fig Molecular landscape and validation of LUSC CSs. (A) Heatmap displaying the expression of immune checkpoint genes and the infiltration of immune cells among the four CS subtypes in the TCGA-LUSC cohort, as assessed by single-sample gene set enrichment analysis (ssGSEA). The top panel shows the expression of canonical immune checkpoint genes, while the bottom panel illustrates the enrichment levels of 28 immune cell types related to tumor microenvironment (TME). (B) Heatmap of metabolism-related pathways among the four CS subtypes in the TCGA-LUSC cohort by ssGSEA. (C) Regulon activity profiles for potential regulators associated with chromatin remodeling (top) and TFs (bottom) across the four CSs. (D) Validation of CSs using the nearest template in the GEO-LUSC cohort. (E) Consistency of CSs with chromatin remodeling (NTP) in the TCGA-LUSC cohort.
(PDF)

S2 Fig Low-LUSCSPI group showed higher mutation frequencies. (A) Mutation profiles of genes in the LUSCSPI model between high- and low- LUSCSPI patients. (B) Mutation profiles of high-frequency mutated LUSC genes in high- and low-LUSCSPI patients.
(PDF)

S3 Fig Correlation between oncogene/suppressor genes and LUSCSPI. (A) Differential expression of oncogene/suppressor genes in high- and low-LUSCSPI patients. (B) Correlation analysis between cancer-related gene expression and LUSCSPI.
(PDF)

Table S1 Twenty genes used for LUSC prognostic signature along with their corresponding coefficients. (XLSX)

Table S2 Clinical information of LUSC patients. (CSV)

Table S3 Abundance of 28 immune cell types based on the ssGSEA algorithm between high- and low-LUSCSPI patients. (CSV)

Table S4 Expression of immune-related, immune checkpoints-related, and HLA-related genes between high- and low-LUSCSPI patients. (CSV)
