# Supplementary figures and images for: A novel prognostic model for lung squamous cell carcinoma based on multi-omics analysis and machine learning
Source: PLoS One. 2025 Dec 19;20(12):e0336792. doi: 10.1371/journal.pone.0336792 (PMC12716744; doi:10.1371/journal.pone.0336792)

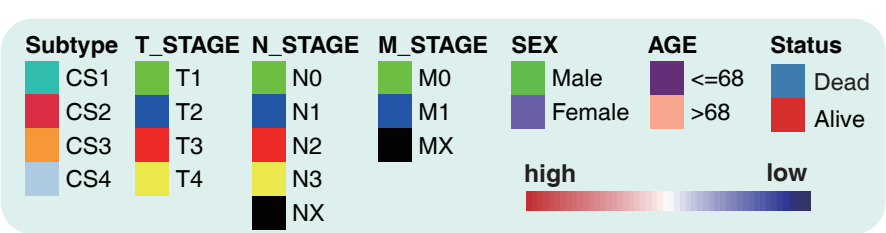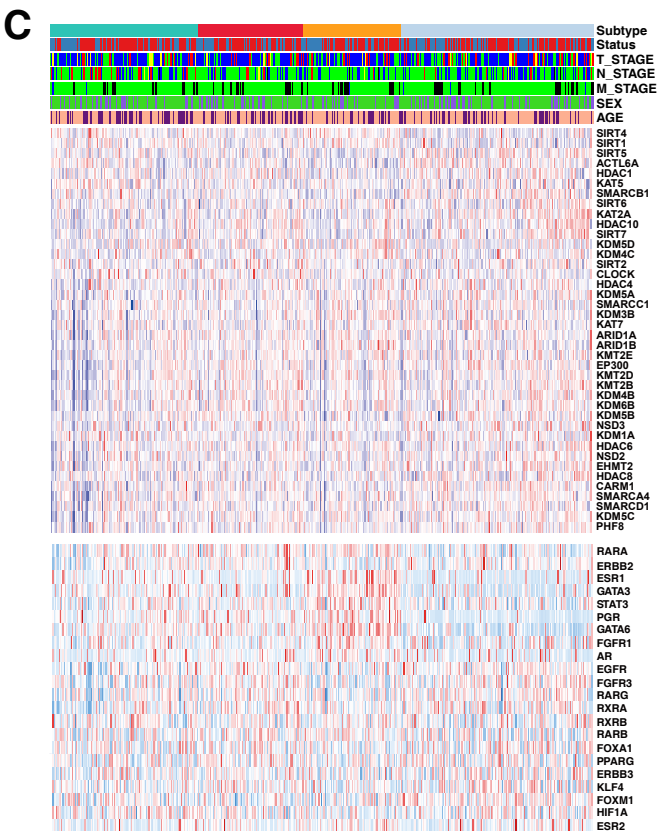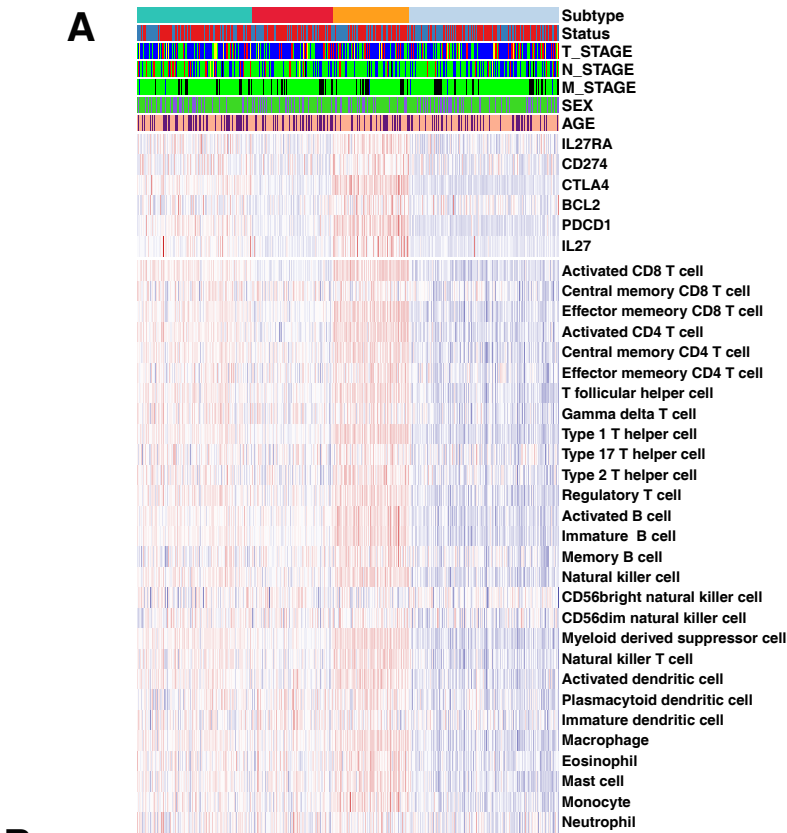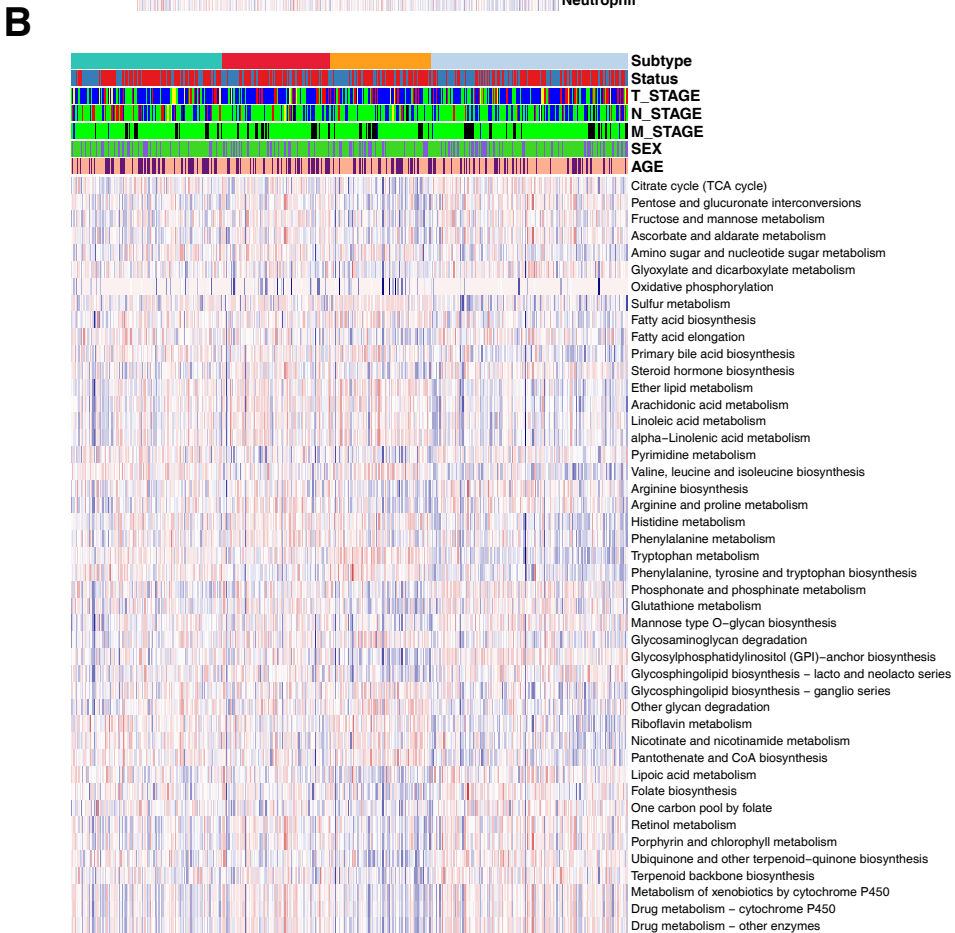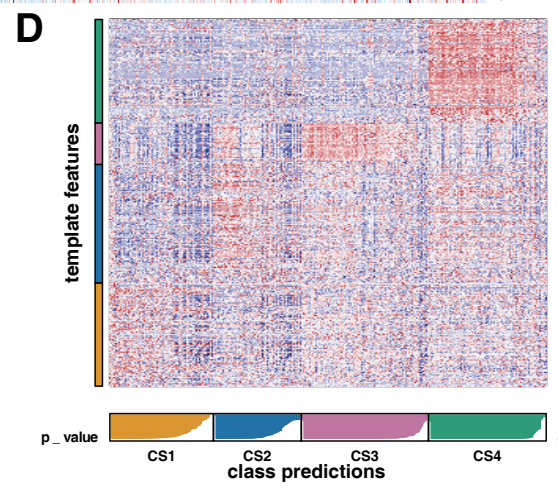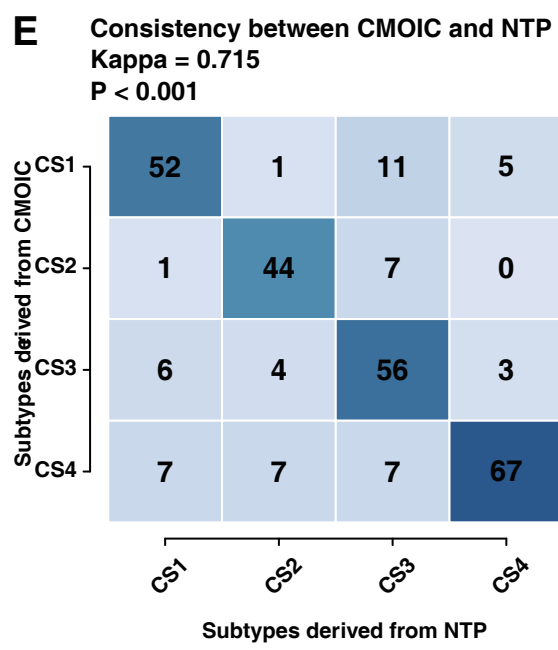

Supplement: S1 Fig — (A) Heatmap displaying the expression of immune checkpoint genes and the infiltration of immune cells among the four CS subtypes in the TCGA-LUSC cohort, as assessed by single-sample gene set enrichment analysis (ssGSEA). The top panel shows the expression of canonical immune checkpoint genes, while the bottom panel illustrates the enrichment levels of 28 immune cell types related to tumor microenvironment (TME). (B) Heatmap of metabolism-related pathways among the four CS subtypes in the TCGA-LUSC cohort by ssGSEA. (C) Regulon activity profiles for potential regulators associated with chromatin remodeling (top) and TFs (bottom) across the four CSs. (D) Validation of CSs using the nearest template in the GEO-LUSC cohort. (E) Consistency of CSs with chromatin remodeling (NTP) in the TCGA-LUSC cohort. (PDF) [file pone.0336792.s001.pdf]

A

High-LUSCSPI

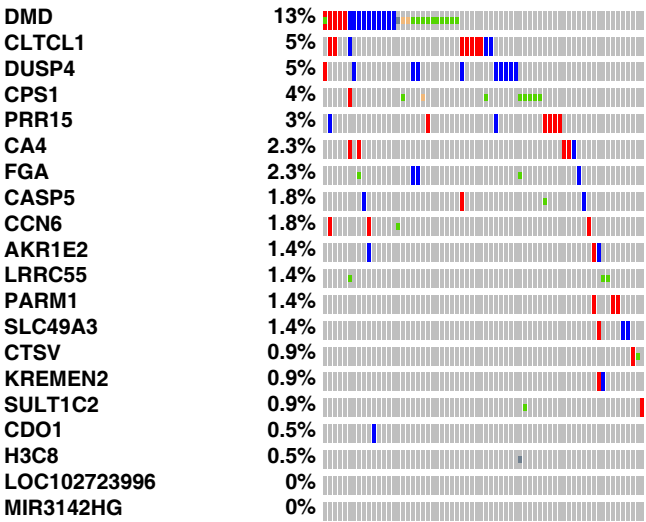

Low-LUSCSPI

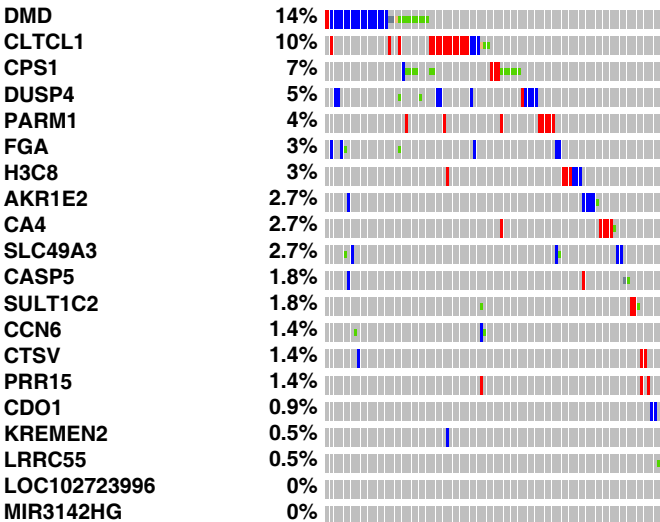

B

High-LUSCSPI

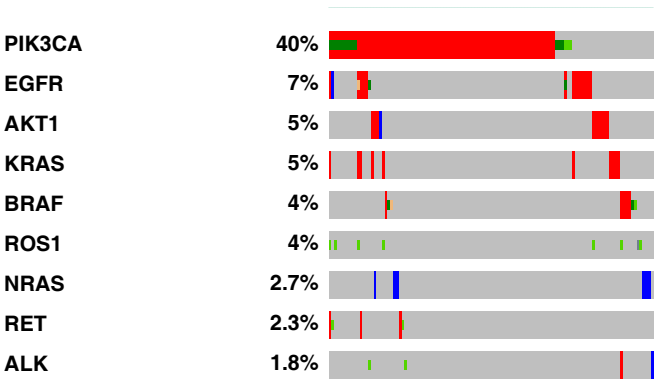

Low-LUSCSPI

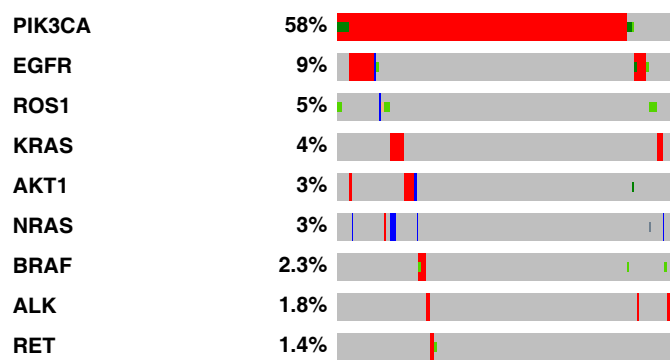

Supplement: S2 Fig — (A) Mutation profiles of genes in the LUSCSPI model between high- and low- LUSCSPI patients. (B) Mutation profiles of high-frequency mutated LUSC genes in high- and low-LUSCSPI patients. (PDF) [file pone.0336792.s002.pdf]

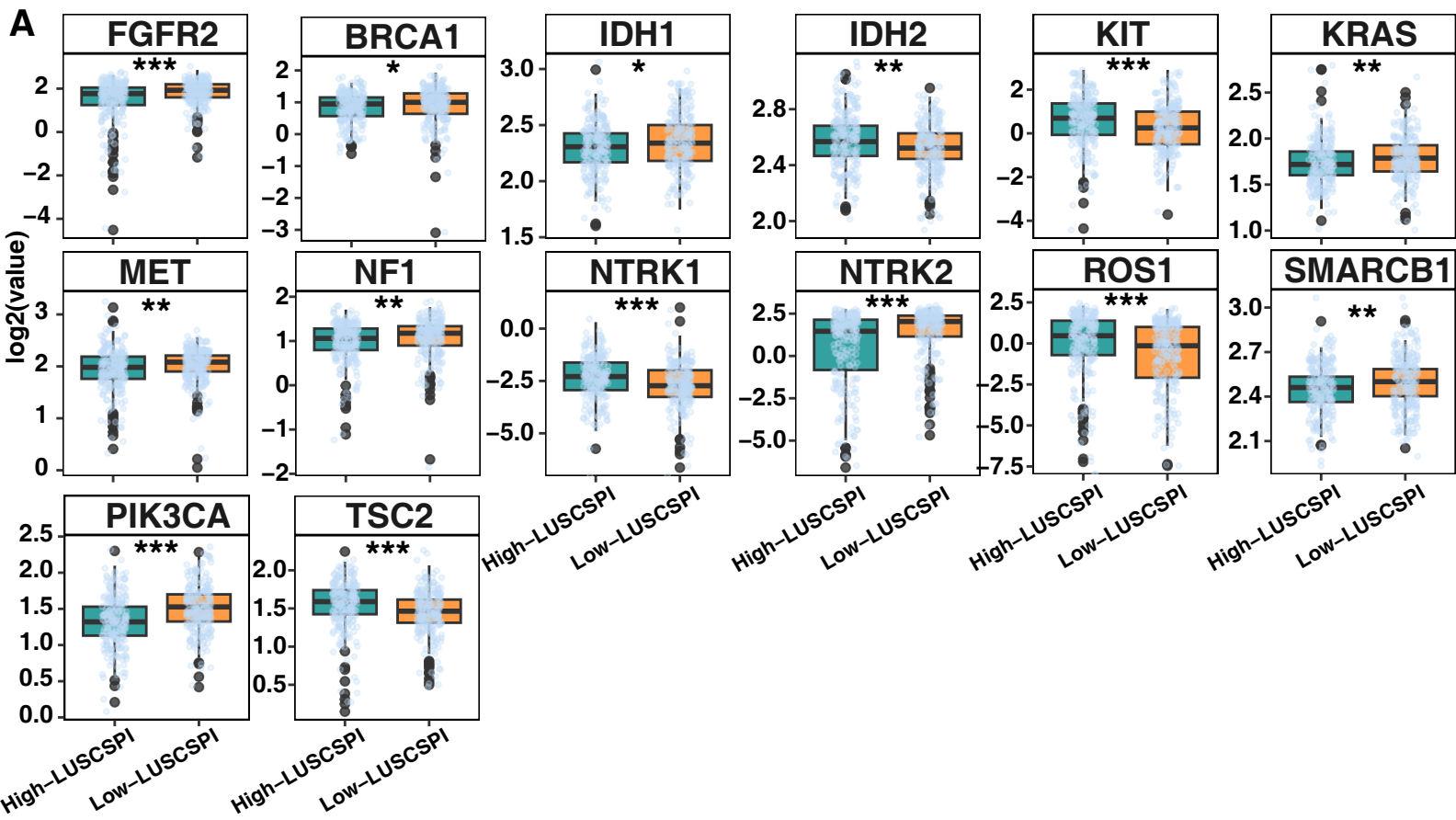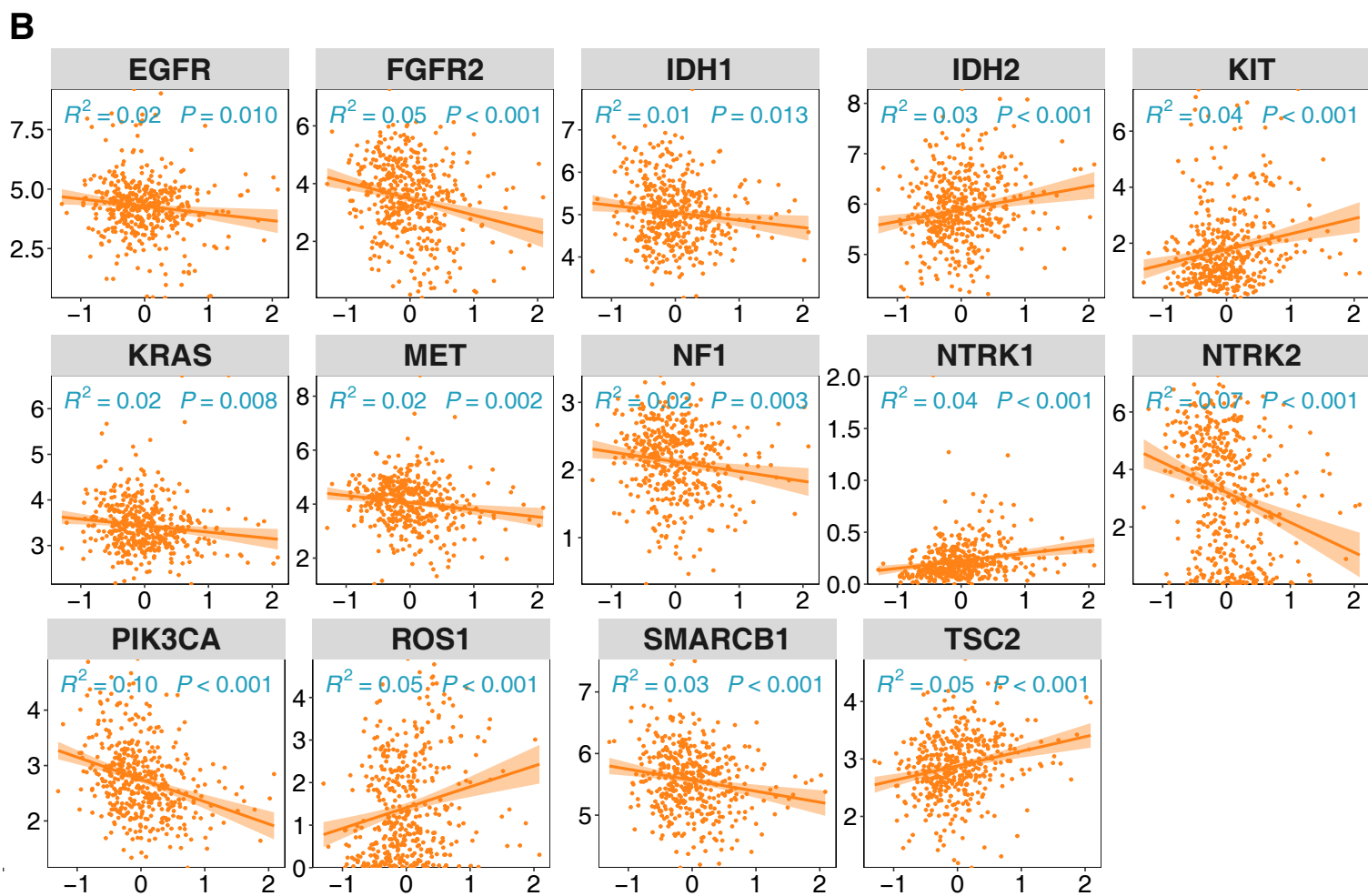

Supplement: S3 Fig — (A) Differential expression of oncogene/suppressor genes in high- and low-LUSCSPI patients. (B) Correlation analysis between cancer-related gene expression and LUSCSPI. (PDF) [file pone.0336792.s003.pdf]
